# Multi-feed, loop-dipole combined dielectric resonator antenna arrays for human brain MRI at 7 T

**DOI:** 10.1007/s10334-023-01078-y

**Published:** 2023-04-05

**Authors:** Daniel Wenz, Thomas Dardano

**Affiliations:** 1 CIBM Center for Biomedical Imaging, École Polytechnique Fédérale de Lausanne (EPFL), Lausanne, Switzerland; 2 Animal Imaging and Technology, École Polytechnique Fédérale de Lausanne (EPFL), Lausanne, Switzerland

**Keywords:** Dielectric resonator, Dipole antenna, RF coil, Multi-channel array, Brain MRI 7 T

## Abstract

**Objective:**

To determine whether a multi-feed, loop-dipole combined approach can be used to improve performance of rectangular dielectric resonator antenna (DRA) arrays human brain for MRI at 7 T.

**Materials and methods:**

Electromagnetic field simulations in a spherical phantom and human voxel model “Duke” were conducted for different rectangular DRA geometries and dielectric constants ε_r_. Three types of RF feed were investigated: loop-only, dipole-only and loop-dipole. Additionally, multi-channel array configurations up to 24-channels were simulated.

**Results:**

The loop-only coupling scheme provided the highest B_1_^+^ and SAR efficiency, while the loop-dipole showed the highest SNR in the center of a spherical phantom for both single- and multi-channel configurations. For Duke, 16-channel arrays outperformed an 8-channel bow-tie array with greater B_1_^+^ efficiency (1.48- to 1.54-fold), SAR efficiency (1.03- to 1.23-fold) and SNR (1.63- to 1.78). The multi-feed, loop-dipole combined approach enabled the number of channels increase to 24 with 3 channels per block.

**Discussion:**

This work provides novel insights into the rectangular DRA design for high field MRI and shows that the loop-only feed should be used instead of the dipole-only in transmit mode to achieve the highest B_1_^+^ and SAR efficiency, while the loop-dipole should be the best suited in receive mode to obtain the highest SNR in spherical samples of similar size and electrical properties as the human head.

## Introduction

Ultrahigh field (B_0_ ≧ 7 T) MRI offers substantial signal-to-noise (SNR) gain in comparison with standard clinical MRI scanners operating at 1.5 T or 3 T [1]. This gain is particularly desirable by neuroscientists who explore the anatomy, function, connectivity and chemical composition of the human brain in vivo at the macroscopic scale [2]. However, the shorter electromagnetic wavelength at 7 T (about 12 cm in human brain) leads to radio frequency (RF) field non-uniformities, and, consequently, to apparent signal dropouts in MR images [3]. Moreover, specific absorption rate (SAR), which is a measure of RF power deposition in human tissue, increases with the Larmor frequency [4]. To tackle these challenges, multi-channel RF coil arrays are widely used; by controlling the relative RF phase in each channel, a constructive RF interference, e.g. in the center of the human brain can be obtained, resulting in an efficient and locally homogeneous RF field [5]. RF technology development at UHF has been an active area of research in recent years, resulting in several novel designs for neuro applications (e.g. stripline resonators [6], dipole antennas [7, 8] or folded dipole antennas [9]).

Traditional multi-channel RF coil arrays are constructed using conductive loop elements with lumped components (capacitors, inductors) necessary for tuning, matching, and decoupling. There is, however, a promising alternative which requires no additional decoupling circuits and only a minimal number of lumped components: the dielectric resonator antenna (DRA) [10–13]. DRAs can be manufactured from a dielectric material (e.g. ceramic) of desired geometry and electrical properties (dielectric constant ε_r_ and loss tangent tanδ) [14]. By using different RF coupling schemes (e.g. a small loop element), different time-invariant electromagnetic field patterns, called modes, can be excited within the dielectric block [15, 16]. For instance, electric $\overrightarrow{E}$ and magnetic field $\overrightarrow{H}$ distribution for some transverse electric (TE) modes can provide not only an MR-efficient transmit field (B_1_^+^) but also high inter-element isolation in a multi-channel DRA [17]. The latter eliminates the need for using additional decoupling methods (overlapping, inductive or capacitive) which are required in standard loop coil arrays and which fix the array’s elements with respect to each other. This aspect can be considered suboptimal for emerging neuroimaging techniques in which 7 T MRI is combined with other modalities, e.g. electroencephalography—EEG [18]. Most of the available, commercial loop coil arrays are not designed for multi-modal imaging, resulting in the array being either too “spacious” or too tight (especially if an additional multi-channel receive-only array is used). In this context, DRA arrays can provide much more flexibility; due to low inter-channel coupling, each element of a DRA array can be freely positioned around the head, thereby providing an opportunity to improve transmit (and receive) performance for given experimental conditions.

Interestingly, in previous studies when a loop element was used as an RF feed, dielectric modes were thoroughly investigated and dielectric blocks were interpreted as loop-coupled dielectric resonators antennas [10, 11, 13, 17]. This stands in contrast with other reports in which the loop element was replaced with a dipole antenna immersed in (or placed on the top/bottom of) a dielectric block [19–23]. Those reports focused on the dipole antenna design, and the influence of dielectric modes on the transmit field distribution was not emphasized. However, a recent study of ours showed that dipole antennas, which are in contact with a dielectric block, act as dipole-fed dielectric resonator antennas [24]. They can couple, just like loops, to different types of TE modes formed within rectangular blocks (especially if the cutoff frequency for a given dielectric mode is below the Larmor frequency), and such modes have a major impact on the B_1_^+^ distribution.

If the “DRA interpretation” is correct, and both loop and dipole elements can excite different dielectric modes, there is still a critical, unresolved issue: which one of them should be used to maximize transmit (and receive) performance of a DRA? While it was shown that a dipole-fed rectangular DRA can provide good transmit efficiency in deeper located regions, e.g. prostate [19, 25], it is unknown whether the same is true in the context of human brain MRI at 7 T. The human head is a significantly smaller anatomical structure, and its size could be a factor limiting the benefit from the “far-field effect” at 300 MHz which is much more pronounced for body MRI [19]. Moreover, the interaction between a given dielectric mode and a sample depends on the sample’s geometry [24]. This means that any findings reported for a large rectangular phantom, mimicking human body, cannot be directly applied in the context of the human brain, which is closer to a spherical geometry.

Multi-channel dielectric resonator antennas for brain MRI at 7 T were studied by Winter et al. [20] who developed an 8-channel bow-tie antenna array. A single building block of that array was constructed using a bow-tie antenna element immersed in deuterium oxide (D_2_O). Their recent work focused on increasing the number of elements by using smaller dielectric blocks with higher ε_r_ value [23]. It can be observed that in configurations with a higher number of elements (e.g. 16 or 24), inter-element coupling can be problematic, and additional RF shields were used in their simulations. A similar approach was proposed by Sadeghi-Tarakameh et al. who showed that combining dipole antennas with optimized dielectric blocks can be a promising solution for MRI at 10.5 T [26].

So, even though some practical dipole-fed rectangular DRA designs for brain MRI have been proposed, it still remains unknown whether they can provide an optimal transmit (and receive) performance for brain MRI at 7 T. It is also unclear which dielectric constant and dielectric mode should be considered for such an application. By addressing these issues, we could enhance our understanding of DRA for UHF-MRI applications, and, potentially, achieve higher transmit (and receive) performance. This is particularly interesting in the context of the recent work [27], demonstrating that by using a combined loop-dipole coupling scheme, a substantial B_1_^+^ efficiency (35% in the center) gain can be achieved compared to a dipole-only coupling scheme for a 16-channel rectangular DRA. That study provided preliminary evidence that the dipole antenna alone might not be the most favorable coupling scheme for rectangular DRA in human brain MRI at 7 T.

The goal of this study, therefore, was to: (a) determine which type of RF feed (loop-only, dipole-only or loop-dipole) for a rectangular DRA can provide the highest transmit and receive performance in the context of human brain MRI at 7 T, and (b) how these findings can be translated into novel multi-feed, loop-dipole combined DRA arrays for human brain MRI at 7 T.

## Materials and methods

### Electromagnetic field simulations

Numerical electromagnetic field and SAR simulations were performed using the finite-difference time-domain solver of Sim4Life (Zurich Medtech, Zurich, Switzerland). Simulations were either performed in a spherical phantom (radius = 85 mm, ε_r_ = 56, σ = 0.66 S/m) or the human voxel model “Duke” from the Virtual Family [28]. Copper elements were modeled as perfect electrical conductors (PEC). The excitation signal was of Gaussian type (center frequency = 297.2 MHz and bandwidth = 500 MHz). The grid was manually adjusted for all the components in the simulation. The smallest mesh cell was 2 mm (“Duke”, conductors, dielectric blocks, ports), while for the spherical phantom it was 4 mm. An RF shield was not included in the simulations. For each multi-port simulation, the data were exported as Touchstone files and used in Advanced Design System (Keysight Technologies, CA, USA) to design the tuning/matching RF circuits. Subsequently, the same RF circuits were reproduced in the MATCH module of Sim4Life. The combined electromagnetic fields were calculated and the results were normalized to 1 W input power. Transmit field efficiency was defined as B_1_^+^/√P, where P is the input power, and SAR efficiency defined as B_1_^+^/√SAR_10g_, where SAR_10g_ is the maximum SAR averaged over 10 g. Signal-to-noise ratio (SNR) was evaluated using an implementation of the Roemer’s algorithm [29] which was based on the S-matrix formalism proposed by Kuehne et al. [30, 31].

### Dielectric resonator antenna (DRA)

**Fig. 1:**
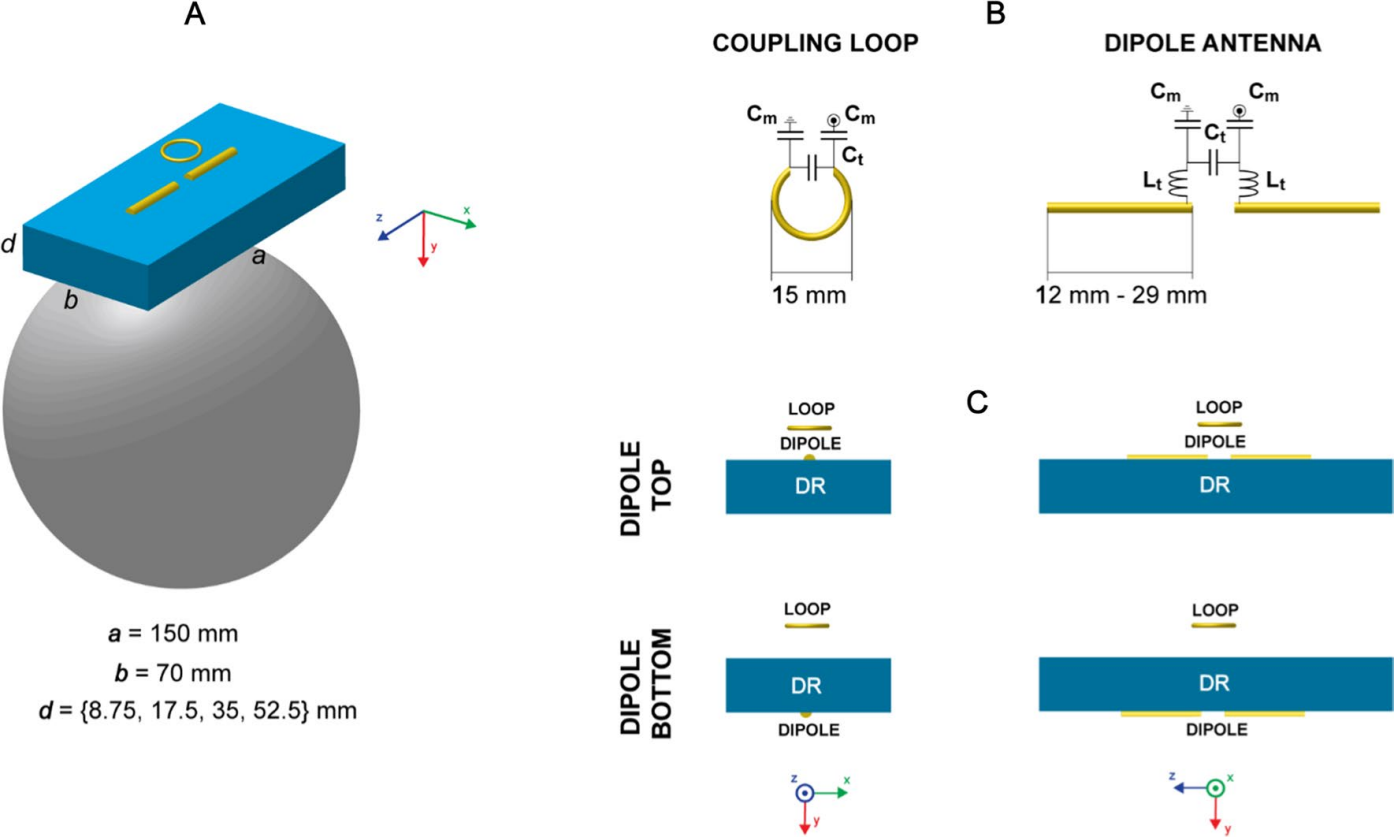
**A** Rectangular dielectric resonator antenna (*a* = 150 mm, *b* = 70 mm, *d* = {8.75 mm, 17.5 mm, 35 mm, 52.5 mm}) loaded with a spherical phantom (radius = 85 mm). A set of different dielectric constants ε_r_ from 75 to 400 was investigated. A small loop element was combined with a dipole antenna and used as an RF feed. **B** Coupling loop and dipole antenna connected to their corresponding tuning/matching circuits (C_t_—tuning capacitor, C_m_—matching capacitor, L_t_—tuning inductor). The diameter of the loop was constant (15 mm) and the length of the dipole antenna depended on dielectric permittivity of the rectangular block. **C** Two different positions of dipole antenna with respect to dielectric resonator antenna were investigated: top and bottom. The distance between the loop and the dielectric block was constant and equal to 15 mm

Previous work [24] provided the motivation to investigate performance of transverse electric (TE) modes other than ${TE}_{11\delta }^{z}$ induced in rectangular dielectric resonator antenna (DRA). The rectangular geometries included in the analysis were, therefore, chosen to allow for propagation of higher-order TE modes. The cutoff frequency for given TE mode can be calculated using the following equation:

1 $${f}_{cutoff}=\frac{c}{2\pi \sqrt{{\epsilon }_{r}}}\sqrt{{\left(\frac{\pi m}{a}\right)}^{2}+{\left(\frac{\pi n}{b}\right)}^{2}+{\left(\frac{\pi l}{d}\right)}^{2}},$$

where *c* is the velocity of light, *m*, *n*, *l* are the dielectric mode indices ($\delta$ means that there is a fraction of a field half-cycle in the given direction) and *a*, *b*, *d* are the rectangular block dimensions according to Fig. 1. The types of different modes can be determined based on the observed three-dimensional electromagnetic field pattern inside each block using the convention presented elsewhere [32]. Index *m* refers to the electromagnetic field pattern along the longest axis of the block, *n* to the width of the block, and *l* to the height of the block. To enable propagation of higher order modes (especially for higher ε_r_ values), the length *a* (150 mm) and the width *b* (70 mm) of a rectangular dielectric block were constant while the height *d* varied as follows: 0.125*b* = 8.75 mm, 0.25*b* = 17.5 mm, 0.5*b* = 35 mm, 0.75*b* = 52.5 mm. For such defined dielectric block geometries, a set of dielectric constants ε_r_ was investigated: (75, 100, 125, 150, 175, 200, 225, 250, 275, 300, 350, 400). The dimensions were also chosen so that the block could be used as one of the elements in a close-fitting 8-channel transmit/receive array for brain imaging. The dimensions and ε_r_ of each block were not designed to exactly match the resonance condition for given dielectric mode and the Larmor frequency. However, different TE modes, according to the Eq. 1, were allowed to propagate and had a critical impact on the DRA’s performance [24]: ${f}_{cutoff}$ for ${TE}_{110}$ is lower than the Larmor frequency for each one of the analyzed rectangular blocks. Moreover, for such a set of dimensions (depending on the ε_r_ value), higher-order TE modes could be also induced (maximum individual indices: *m* = 5, *n* = 2, *l* = 2). To define electrical conductivity σ for each block, the one with distilled water at 300 MHz (σ = 0.02 S/m) was used as the baseline value. To keep tanδ constant in each simulation, σ was calculated for each ε_r_ value according to the following equation: tanδ = σ/ωε, where ω is the angular frequency, and ε = ε_r_ε_0_ where ε_0_ is the vacuum permittivity. This resulted in the following σ values (in S/m): 0.0186 (ε_r_ = 75), 0.0248 (ε_r_ = 100), 0.031 (ε_r_ = 125), 0.0372 (ε_r_ = 150), 0.0434 (ε_r_ = 175), 0.0496 (ε_r_ = 200), 0.0558 (ε_r_ = 225), 0.062 (ε_r_ = 250), 0.0682 (ε_r_ = 275), 0.0744 (ε_r_ = 300), 0.0868 (ε_r_ = 350), 0.0992 (ε_r_ = 400). In each simulation a combination of a loop element and a dipole antenna was included (Fig. 1). In this work, we refer to three different coupling schemes (or RF feeds): loop-only, dipole-only and loop-dipole. In this study, whenever the loop-dipole feed was used, there was no phase difference between these two types of elements in TX mode. Furthermore, the dipole antenna was placed in two different positions: top and bottom. When the loop-only RF feed was investigated—the dipole antenna was defined as an open circuit, and vice versa, when the dipole-only RF feed was analyzed—the loop element was considered to be an open circuit. In the case of the loop-dipole, both elements were tuned to the Larmor frequency and matched to 50 Ω. The coupling loop was modeled as a PEC wire with inner radius = 7 mm, wire radius = 0.5 mm. The distance between the loop and the dielectric block (15 mm) was constant for each simulation. The length of the dipole antenna was varied depending on the ε_r_ (in millimeters per arm): 29 (ε_r_ = 75), 25 (ε_r_ = 100), 22 (ε_r_ = 125), 20 (ε_r_ = 150), 19 (ε_r_ = 175), 18 (ε_r_ = 200), 17 (ε_r_ = 225), 16 (ε_r_ = 250), 15 (ε_r_ = 275), 14 (ε_r_ = 300), 13 (ε_r_ = 350), 12 (ε_r_ = 400). The arms of the dipole antenna were separated by 10 mm. Half of the dipole antenna was immersed in the dielectric block, and the other half was in contact with the air. The distance between the dielectric block and the spherical phantom was constant for each simulation (10 mm). A standard capacitive tuning/matching network was used to tune the coupling loop to 297.2 MHz and to match the impedance to 50 Ω. The same network was used to tune and match the dipole antenna, but with additional inductance connected in series, which was necessary for some dielectric blocks with smaller *d*.

### Dielectric resonator antenna array: 16- and 24-channels

A group of 12 dielectric blocks was selected based on the single-block data (combined B_1_^+^ and SAR efficiency), and used in multi-channel array simulations in which 16-channel loop-dipole arrays were investigated (dipole bottom). Dielectric blocks (*d* = 0.75*b*) were considered too large, and they were not further investigated in multi-channel array configurations (excluding ε_r_ = 75 (*d* = 0.75*b*)). Each dielectric block was positioned ~ 10 mm from the surface of Duke’s head. A loop-only coupling scheme was used, and each array was driven in circularly polarized (CP) mode with a phase increment of 45º per element. Therefore, even though the total number of loop-dipole elements was 16, 8 loop elements were used in transmit mode. In receive mode, the loop-dipole coupling scheme was used (16 elements in total). The 8-channel bow-tie antenna array, which was used for comparison in this study, was reproduced from Winter et al. [20]. Finally, the multi-feed, loop-dipole combined approach was demonstrated in a 24-channel configuration (3 RF feeds per block: 1 loop element and 2 dipole antennas (bottom)) for ε_r_ = 275 (*d*/*b* = 0.25) and compared in the receive mode with its 16-channel counterpart. For the 24-channel array, 2 dipole antenna elements were positioned in a way that each outer arm of the antenna was 10 mm from the edge of the dielectric block.

### Reference RF coil arrays

Three reference array designs were simulated: an 8-channel loop coil array (a degenerate birdcage coil), an 8-channel fractionated dipole antenna and an 8-channel folded dipole antenna. The individual elements of the first two of them (loop coil, fractionated dipole) were equally distributed around Duke’s head (diameter = 240 mm); the elements were intentionally chosen to be larger than the DRAs to emulate “spacious” arrays mentioned in the introduction. The folded dipole antenna array had a close-fitting design; the distance between each element and Duke’s head was the same as between Duke’s head and the corresponding DRA’s bottom surface (see: 16-channel DRA arrays).

Each element of the loop coil array (length = 260 mm, copper width = 10 mm) was segmented using 10 capacitors (C = 9.5 pF, C_D_ = 30 pF, C_T_ = 0.6 pF). The C-values were chosen to reduce coupling between the adjacent elements and tune each element to the Larmor frequency. A standard matching circuit (C_M_ between 2.8 and 3.2 pF) was used to match each loop element to 50 Ohm. The 8-channel fractionated dipole antenna array was reproduced from the study of Raaijmakers et al. [33] but using shortened antenna elements (285 mm). A series inductance (between 80 and 90 nH) was used to tune each fractionated dipole antenna to the Larmor frequency; a standard capacitive matching circuit was used to match each antenna to 50 Ohm. The 8-channel folded dipole antenna was reproduced from the previous work of Avdievich et al. [34]; simulations were performed including a local elliptical shield to improve the array’s transmit performance, but without additional passive dipoles.

## Results

**Fig. 2:**
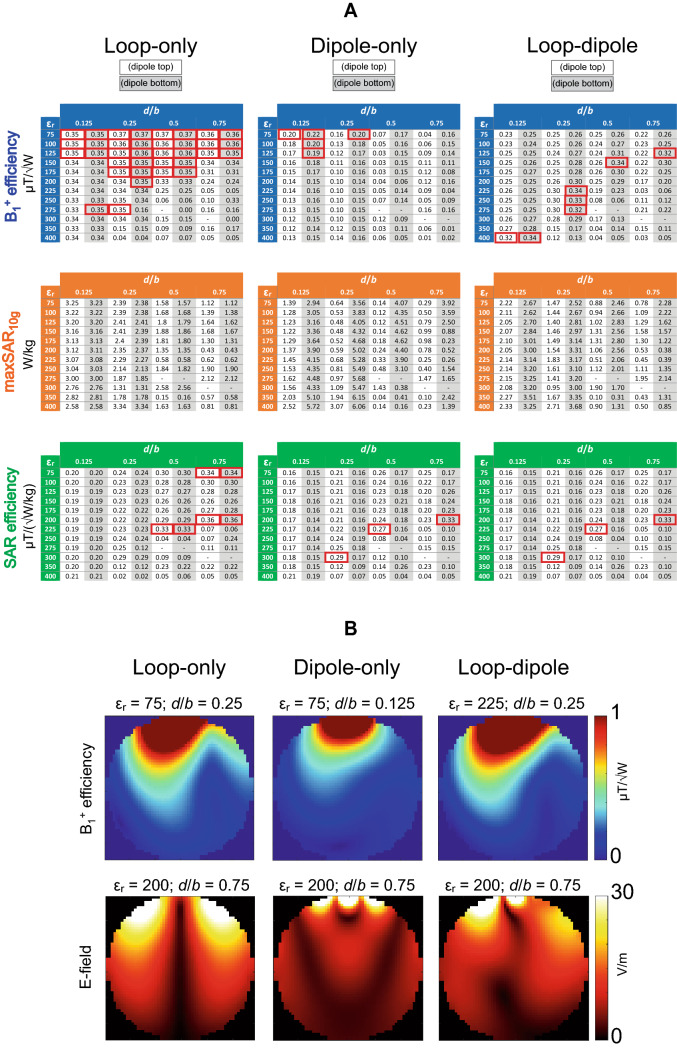
**A** B_1_^+^ efficiency (center), corresponding peak SAR_10g_ and SAR efficiency (center) for the setup from Fig. 1 (for each RF feed type, the red boxes were used to highlight three of the highest B_1_^+^ and SAR efficiencies). A rectangular dielectric resonator antenna was driven in different transmit modes: loop-only, dipole-only and loop-dipole. Each one of these modes was also investigated for two different positions of the dipole antenna: top (cells with white background) and bottom (cells with gray background). For ε_r_ = 275 (*d*/*b* = 0.5) and ε_r_ = 300 (*d*/*b* = 0.75) no data was shown, since it was not feasible to separate coupled dielectric resonances without additional changes in the setup. It is evident that the loop-only coupling scheme outperformed the dipole only and the loop-dipole in terms of B_1_^+^ and SAR efficiency in the vast majority of cases. **B** B_1_^+^ and E-field (absolute) distribution in the central axial slice through the spherical phantom. For each RF feed type, the DRAs which provided the highest B_1_^+^ and SAR efficiency were selected

Electromagnetic field simulations were conducted to investigate which RF coupling scheme (loop, dipole or loop-dipole) provided the highest B_1_^+^ and SAR efficiency in the center of a spherical phantom (radius = 85 mm, ε_r_ = 56, σ = 0.66 S/m) as a function of the dielectric block’s geometry and ε_r_ (Fig. 1). It was found that in the vast majority of cases, the loop-only coupling scheme outperformed dipole-only and loop-dipole in terms of B_1_^+^ and SAR efficiency in the center of the spherical phantom (Fig. 2). The arrangement of the dipole antenna (top or bottom) did not have any significant impact of the performance of the loop-only coupling scheme. Loop-induced modes: ${TE}_{11\delta }^{z}$ and ${TE}_{21\delta }^{z}$ modes provided the highest B_1_^+^ and SAR efficiency. A significant drop in B_1_^+^ efficiency for the loop-only coupling scheme was observed when ${TE}_{21\delta }^{z}$ was transitioning to ${TE}_{31\delta }^{z}$: for *d* = 0.25*b* (when changing ε_r_ from 300 to 350), *d* = 0.5*b* (when changing ε_r_ from 225 to 250) and *d* = 0.75*b* (when changing ε_r_ from 200 to 225). Higher-order loop-induced modes with *m* = 4 and *m* = 5, which were observed in larger blocks with higher ε_r_ (e.g. ε_r_ = 400), showed the lowest transmit performance. The loop-dipole coupling scheme provided significant transmit performance gains vs. the dipole-only. The dipole-only coupling scheme (dipole bottom) provided higher B_1_^+^ efficiency than the dipole-only (dipole top), at the cost of a substantial peak SAR_10g_ increase and reduced SAR efficiency. The difference in transmit performance between dipole-only (top and bottom) was particularly apparent for larger dielectric blocks (*d* = 0.5*b*) in which MR-inefficient modes (dipole top) were induced: ${TE}_{1\delta \delta }^{y}$ and higher ones. The most promising candidates: ε_r_ = 75 (*d* = 0.25*b*), ε_r_ = 75 (*d* = 0.5*b*), ε_r_ = 75 (*d* = 0.75*b*), ε_r_ = 100 (*d* = 0.5*b*), ε_r_ = 125 (*d* = 0.5*b*), ε_r_ = 150 (*d* = 0.5*b*), ε_r_ = 175 (*d* = 0.5*b*), ε_r_ = 200 (*d* = 0.25*b*), ε_r_ = 225 (*d* = 0.25*b*), ε_r_ = 250 (*d* = 0.25*b*), ε_r_ = 275 (*d* = 0.25*b*) and ε_r_ = 300 (*d* = 0.25*b*) were selected for further analysis.

**Fig. 3:**
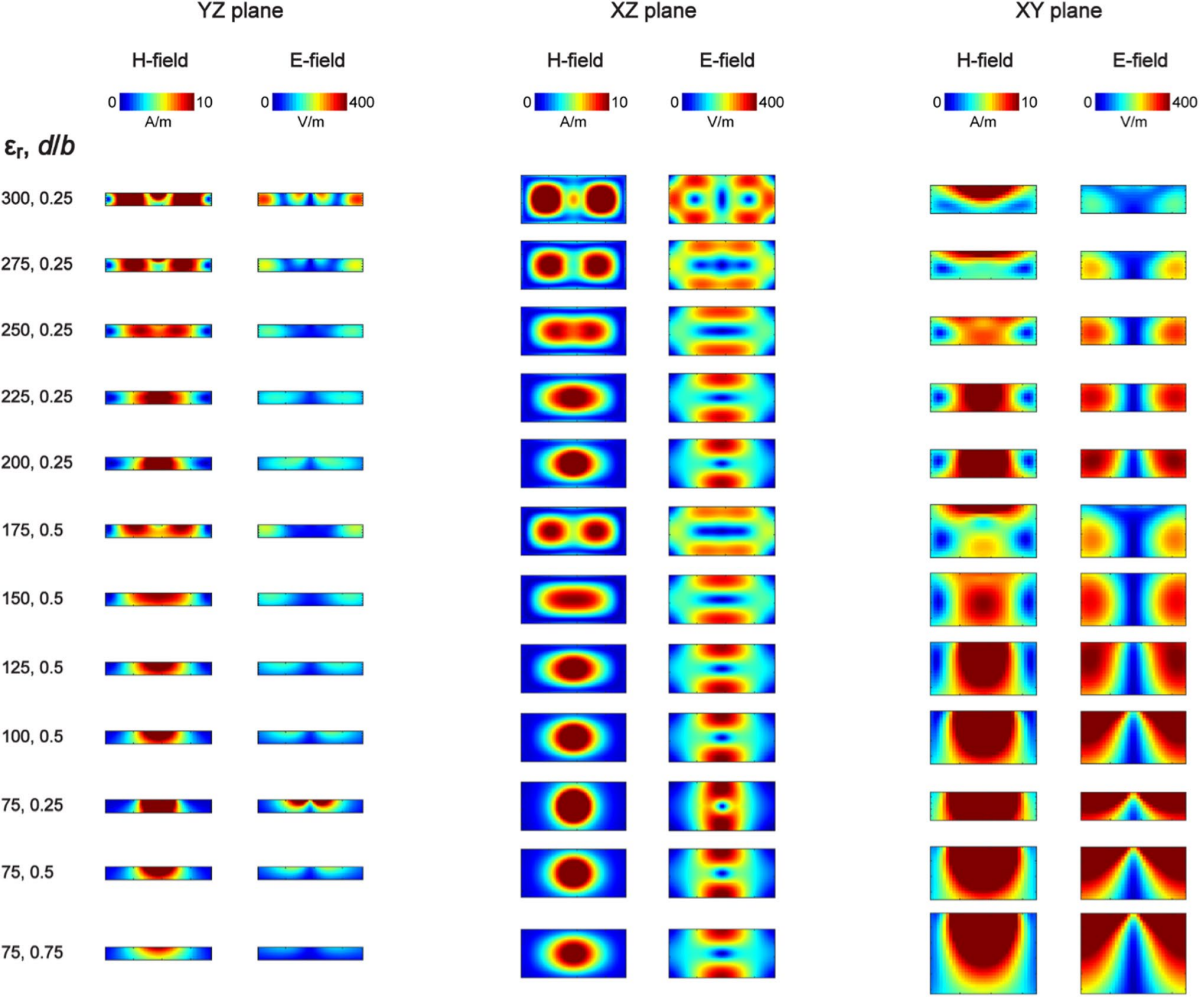
Magnetic $\overrightarrow{H}$ and electric $\overrightarrow{E}$ field distribution inside each one of the selected loop-coupled dielectric resonator antennas. Absolute $\overrightarrow{H}$ and electric $\overrightarrow{E}$ fields are shown in three planes through the central point of each dielectric block. The most apparent difference can be observed in XZ plane with two electromagnetic field maxima for ε_r_ = 175, ε_r_ = 275 and ε_r_ = 300, instead of one as for the other rectangular blocks

To determine which types of dielectric modes were excited in the selected loop-coupled rectangular dielectric resonator antennas, electromagnetic field simulations were performed and the electric $\overrightarrow{E}$ and magnetic field $\overrightarrow{H}$ distributions were obtained for each block (Fig. 3). In most of the cases, there was only one $\overrightarrow{H}$ maximum observed along the z-axis. For three blocks (ε_r_ = 175 (*d* = 0.25*b*), ε_r_ = 275 (*d* = 0.25*b*) and ε_r_ = 300 (*d* = 0.25*b*)), two $\overrightarrow{H}$ field maxima were present along the z-axis. Therefore, dielectric modes (Fig. 2) were identified as transverse electric modes: ${TE}_{11\delta }^{z}$ and ${TE}_{21\delta }^{z}$.

**Fig. 4:**
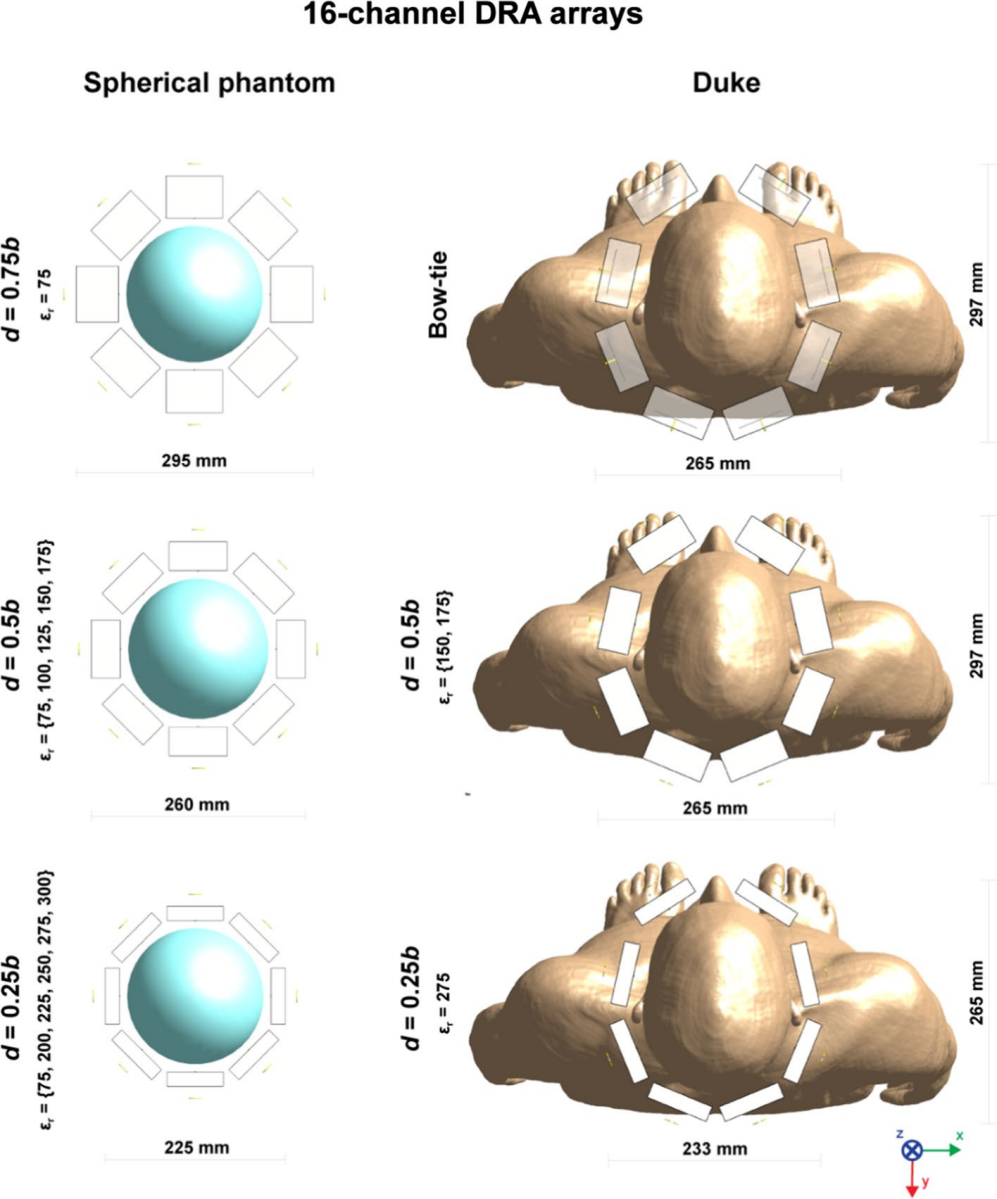
Schematic representations of 16-channel (only bow-tie is an 8-channel array), loop-dipole combined, rectangular dielectric resonator antenna arrays investigated in this study, loaded with a spherical phantom (on the left) and with human voxel model Duke (on the right)

**Fig. 5:**
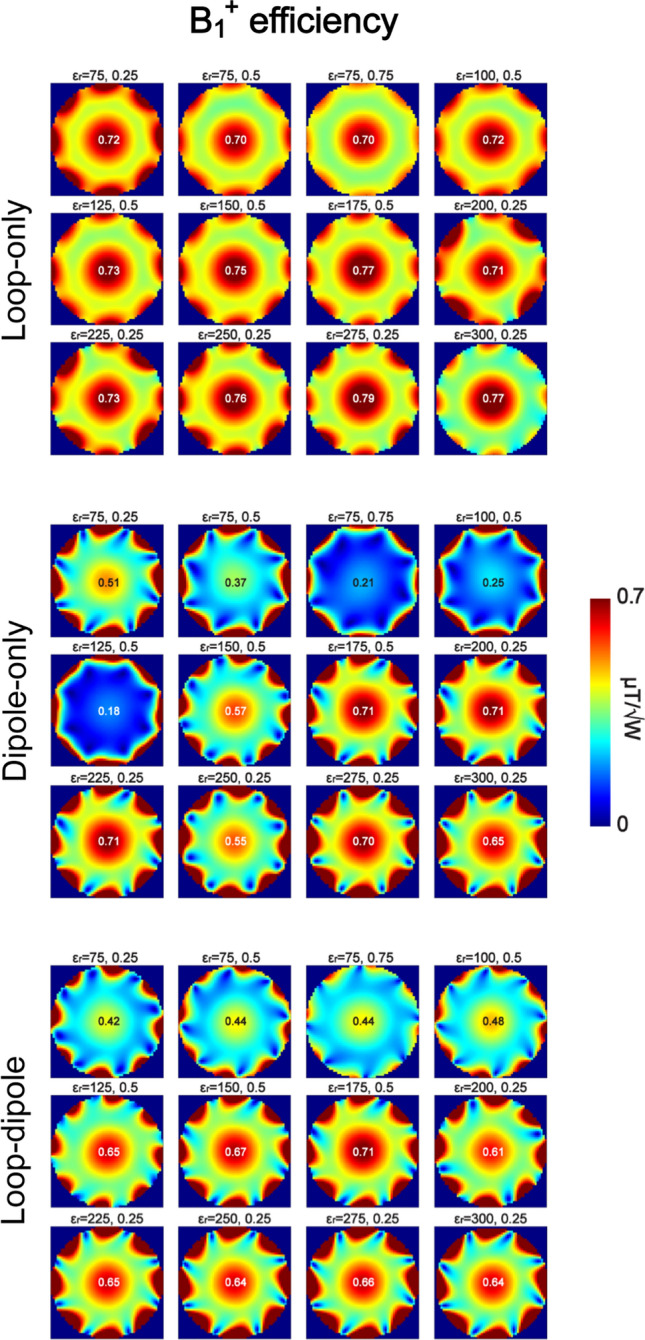
B_1_^+^ distribution in the spherical phantom (central XY plane) for all 12 of the 16-channel, loop-dipole (dipole bottom) coupled dielectric resonator antenna arrays. The data showed that loop-only coupling scheme provided the highest B_1_^+^ efficiency in the center of the phantom for all of the arrays. The highest B_1_^+^ efficiency was observed for the blocks in which a higher-order TE mode was excited: ε_r_ = 275 (0.79 μT/√W), ε_r_ = 175 (0.77 μT/√W) and ε_r_ = 300 (0.77 μT/√W)

**Fig. 6:**
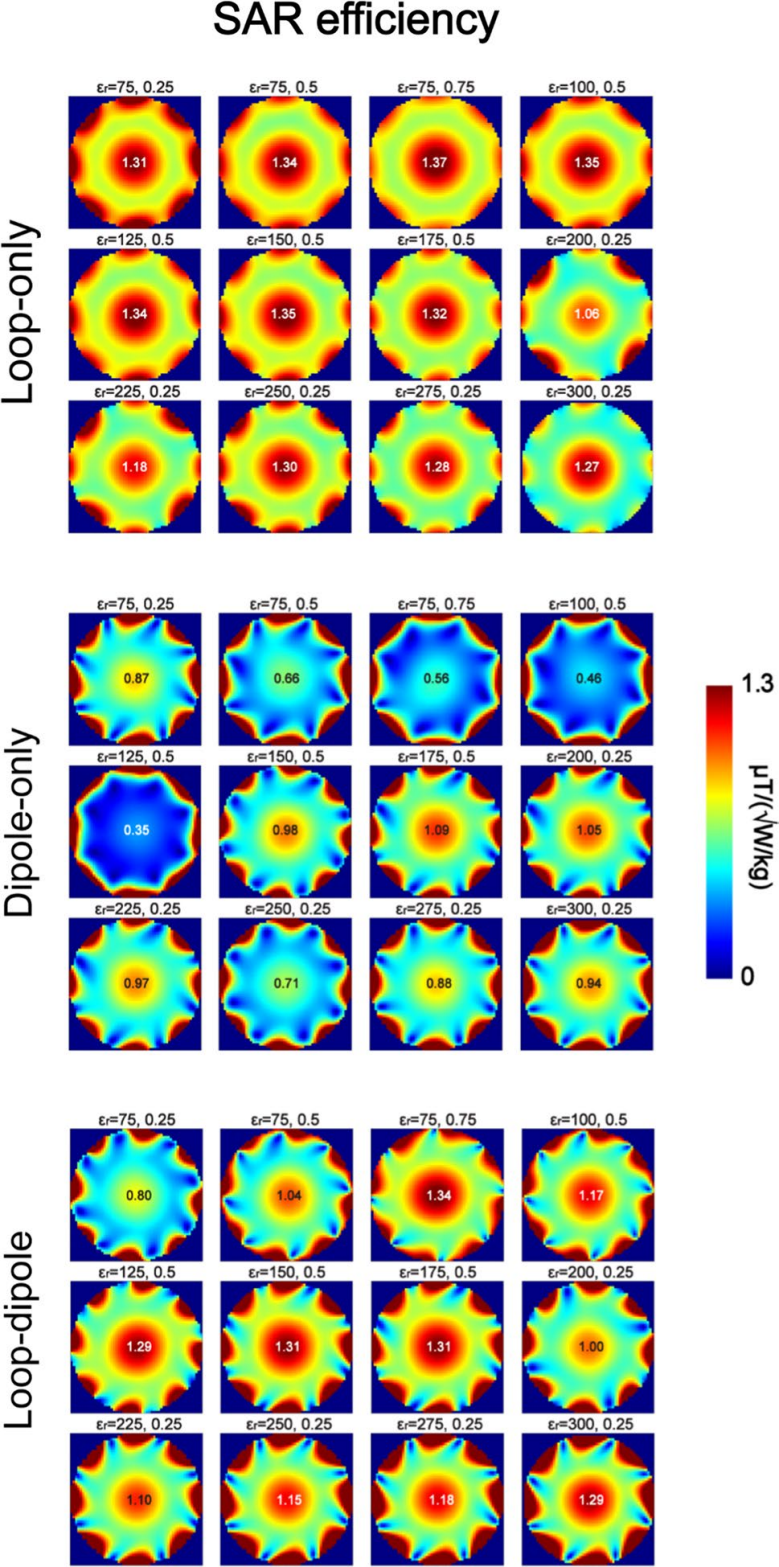
SAR efficiency in the spherical phantom (central XY plane) for all 12 of the 16-channel, loop-dipole (dipole bottom) coupled dielectric resonator antenna arrays. The data showed that loop-only coupling scheme provided the highest SAR efficiency in the center of the phantom for all of the arrays. The highest SAR efficiency was observed for thicker blocks with lower ε_r_ than the ones in Fig. 1: ε_r_ = 75, *d*/*b* = 0.75 (1.37 μT/√(W/kg)); ε_r_ = 100, *d*/*b* = 0.5 (1.35 μT/√(W/kg)) and ε_r_ = 150, *d*/*b* = 0.5 (1.35 μT/√(W/kg))

**Fig. 7:**
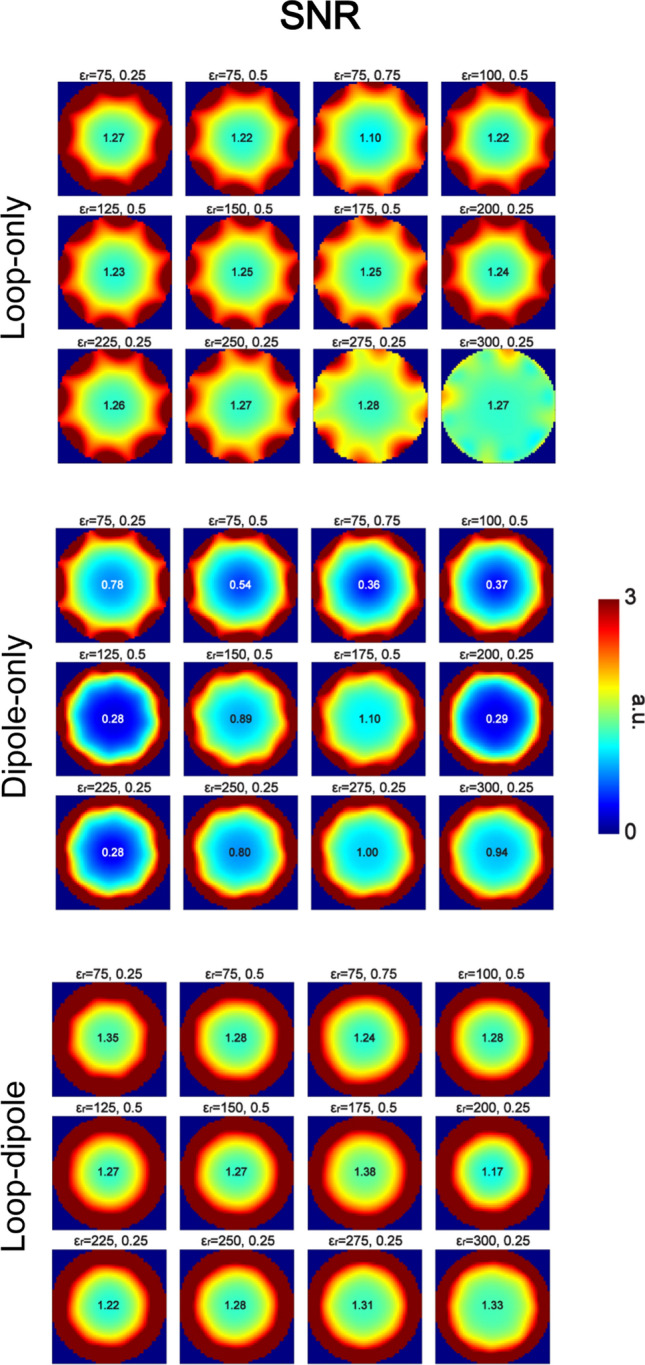
SNR distribution in the spherical phantom (central XY plane) for all 12 of the 16-channel, loop-dipole (dipole bottom) coupled dielectric resonator antenna arrays. The data showed that loop-dipole coupling scheme provided the highest SNR in the center of the phantom for all of the arrays. The highest SNR was observed for the following arrays: ε_r_ = 175, *d*/*b* = 0.75 (1.38 a.u.); ε_r_ = 75, *d*/*b* = 0.25 (1.35 a.u.) and ε_r_ = 300, *d*/*b* = 0.5 (1.33 a.u.)

Electromagnetic simulations were performed to investigate which type of RF feed and which dielectric constant ε_r_ provided the highest transmit and receive performance in an 8-channel (loop-only and dipole-only) and 16-channel (loop-dipole) array configuration (Fig. 4). In all of the analyzed cases, the highest B_1_^+^ and SAR efficiency (Figs. 5, 6) for a circularly polarized (CP) mode was observed for the loop-only coupling scheme, while the highest SNR was observed for the loop-dipole coupling scheme (Fig. 7). The highest B_1_^+^ efficiency (loop-only, Fig. 5) in the center of the spherical was obtained for ε_r_ = 275 (0.79 μT/√W), ε_r_ = 175 (0.77 μT/√W) and ε_r_ = 300 (0.77 μT/√W). The highest SAR efficiency (loop-only, Fig. 6) was obtained for: ε_r_ = 75, *d*/*b* = 0.75 (1.37 μT/√(W/kg)); ε_r_ = 100, *d*/*b* = 0.5 (1.35 μT/√(W/kg)) and ε_r_ = 150, *d*/*b* = 0.5 (1.35 μT/√(W/kg)). The highest SNR (loop-dipole, Fig. 7) was found for the DRA with ε_r_ = 175, *d*/*b* = 0.75 (1.38 a.u.); ε_r_ = 75, *d*/*b* = 0.25 (1.35 a.u.) and ε_r_ = 300, *d*/*b* = 0.25 (1.33 a.u.).

**Fig. 8:**
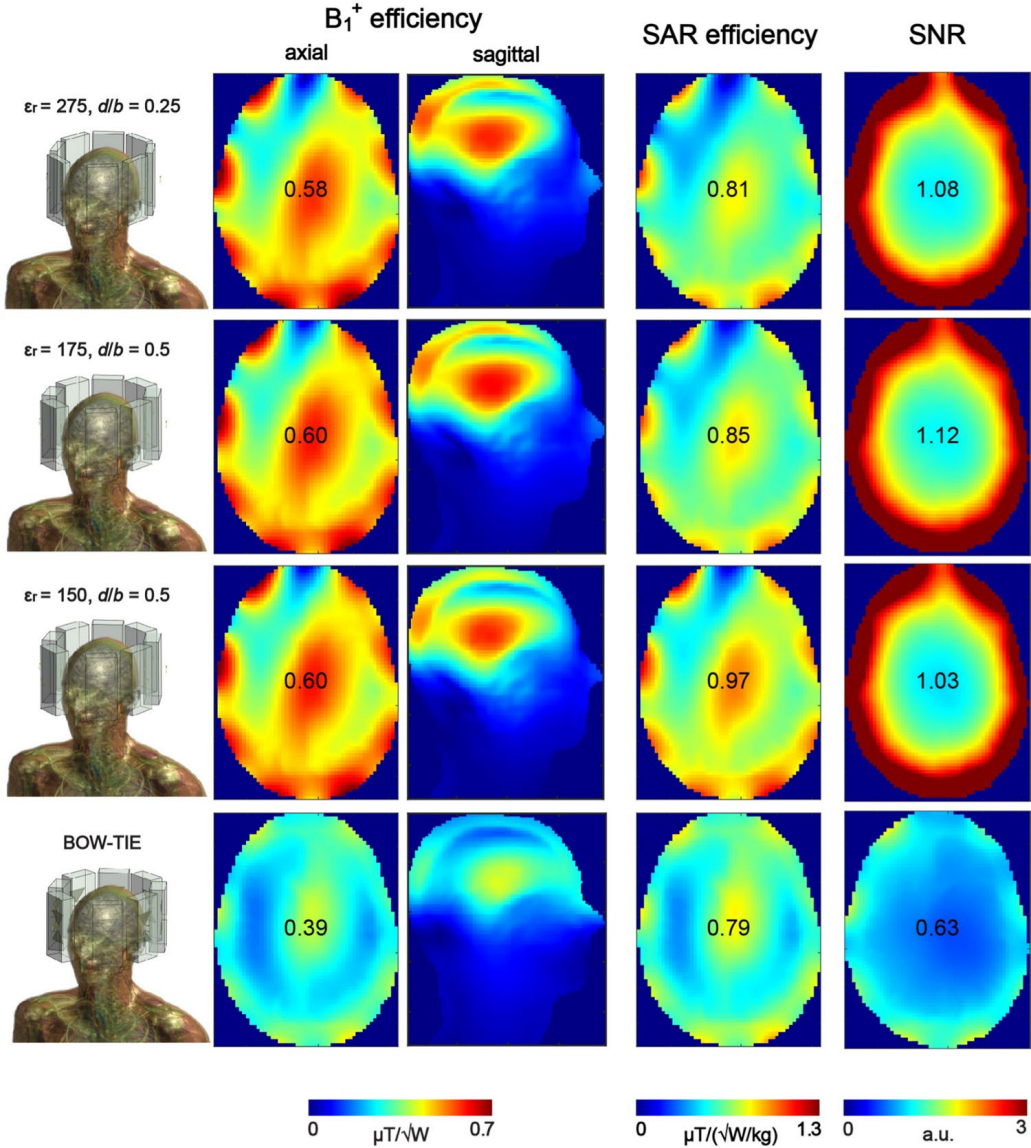
B_1_^+^ efficiency, SAR efficiency and SNR in the human voxel model Duke (central XY plane). Three different loop-dipole coupled rectangular dielectric resonator arrays were chosen and benchmarked against an 8-channel bow-tie antenna array. Each one of the loop-dipole coupled arrays (loop-only for transmission, and loop-dipole for reception) provided substantial B_1_^+^ efficiency (1.54-fold for ε_r_ = 175) and SNR (1.77-fold for ε_r_ = 175) gain in the center. SAR efficiency was also higher especially for ε_r_ = 150 (1.23-fold)

To benchmark the performance of 16-channel loop-dipole combined DRA arrays (in TX mode with 8-channels loop-only and in RX mode with 16 channels loop-dipole) against the state-of-the-art 8-channel bow-tie antenna array, electromagnetic field simulations in Duke were performed (Fig. 8). The data showed that B_1_^+^ efficiency in the center of Duke’s head was higher for all three of the loop-dipole combined arrays (loop-only used for RF transmission): 1.53- (ε_r_ = 150 and 175) and 1.49-fold (ε_r_ = 275). An average B_1_^+^ efficiency across the whole axial slice was evaluated for each DRA array: 0.44 ± 0.10 μT/√W (ε_r_ = 275), 0.45 ± 0.09 μT/√W (ε_r_ = 175), 0.44 ± 0.09 μT/√W (ε_r_ = 150), 0.28 ± 0.05 μT/√W (bow-tie). SAR efficiency was quite similar for ε_r_ = 175 (1.08-fold higher) and for ε_r_ = 275 (1.03-fold higher). The clearest gain in SAR efficiency vs. bow-tie array was observed for ε_r_ = 150 (1.23-fold). There was a substantial gain in the SNR in the center of Duke’s head for all three 16-channel loop-dipole combined arrays vs. bow-tie array with 1.78-fold higher gain for ε_r_ = 175, 1.71-fold for ε_r_ = 275 and 1.63-fold for ε_r_ = 150.

**Fig. 9:**
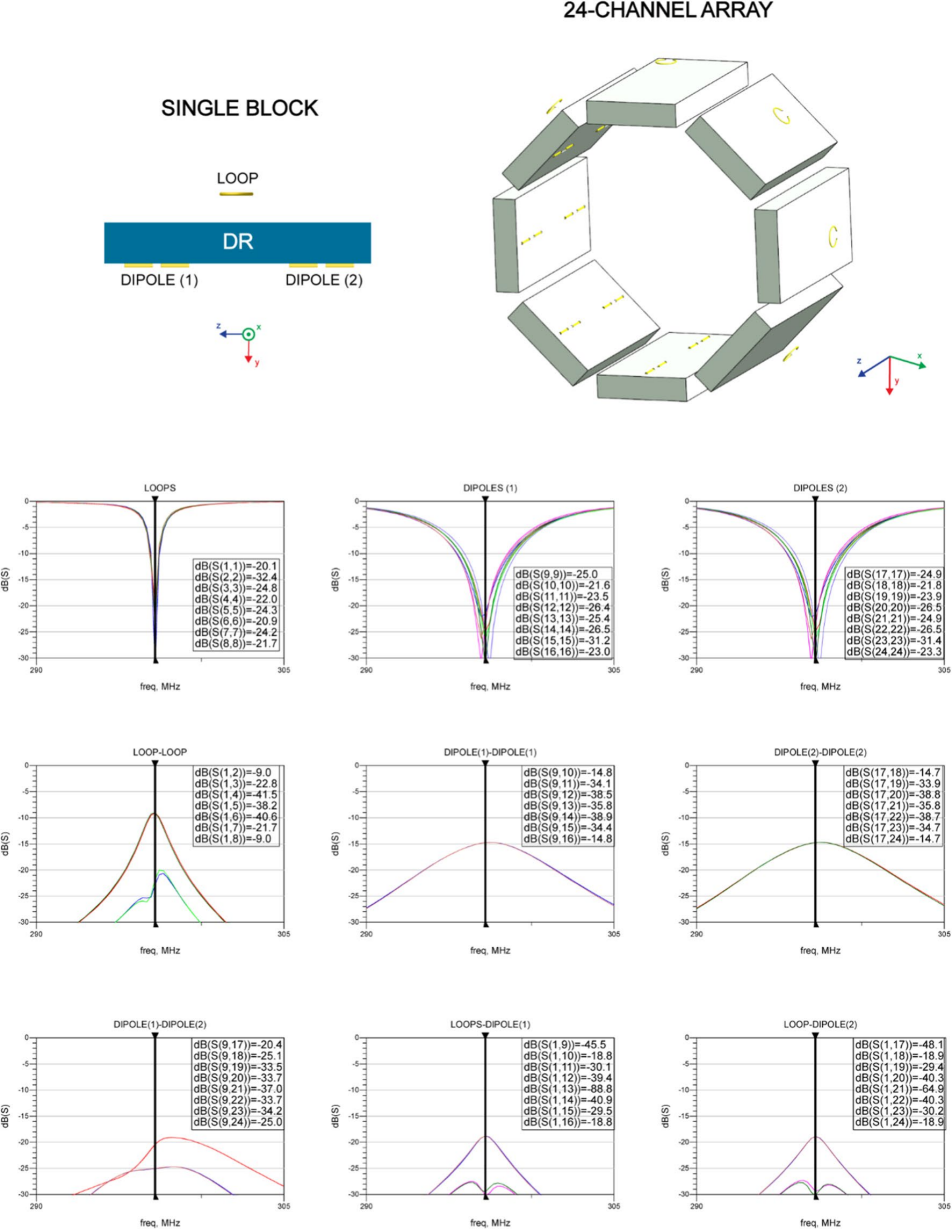
Multi-feed, loop-dipole combined, 24-channel rectangular dielectric resonator antenna array (ε_r_ = 275, *d*/*b* = 0.25). The scattering parameter matrix was evaluated. For the adjacent elements, loop-loop coupling was − 9.0 dB. Dipole–dipole (row 1) coupling was − 14.8 dB and − 14.7 dB (row 2). Dipole–dipole coupling between adjacent rows (1 and 2) was below − 20 dB. Loop-dipole coupling was − 18.8 (row 1) and − 18.9 (row 2)

**Fig. 10:**
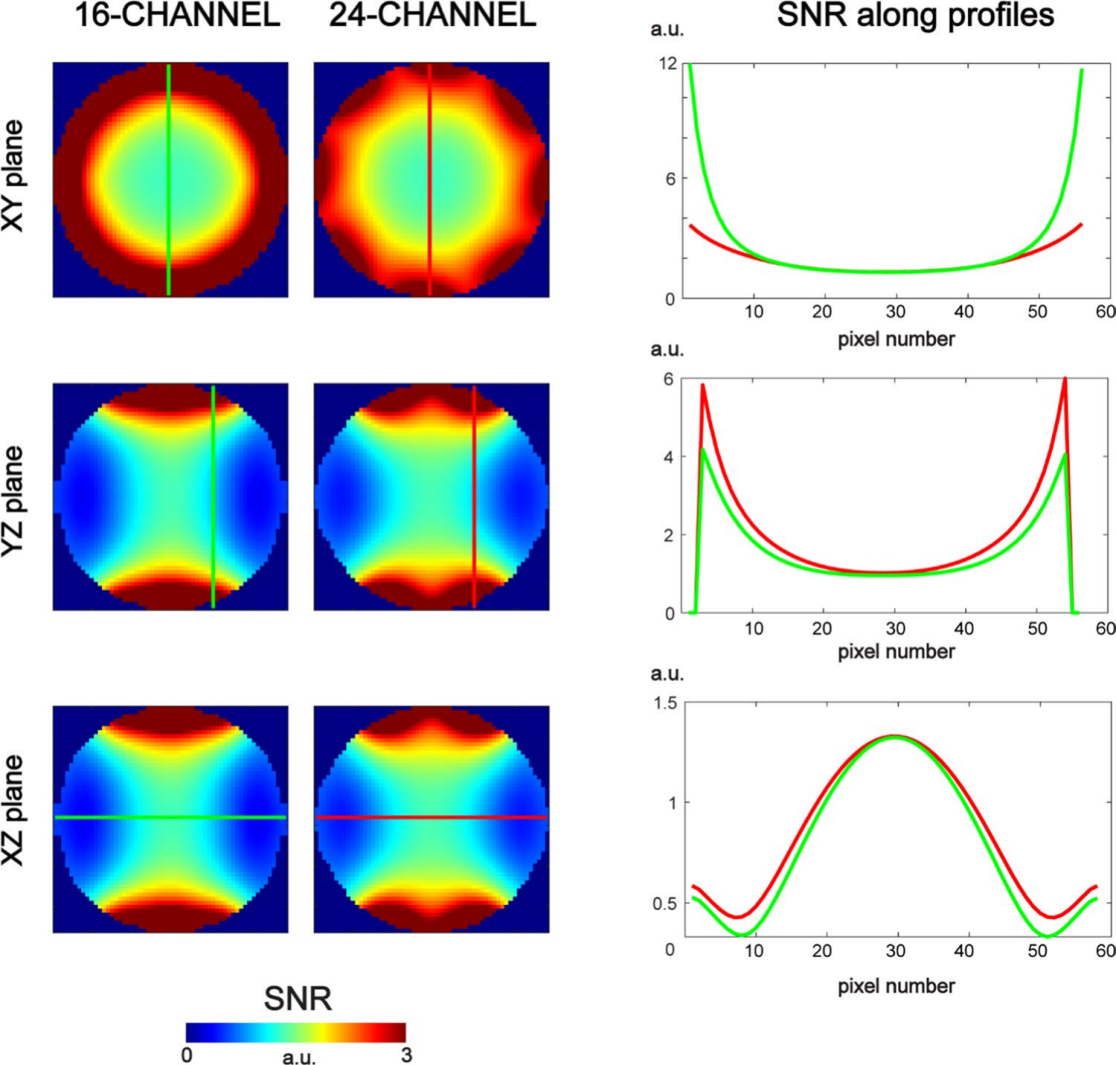
SNR distribution in the spherical phantom for the 16- and 24-channel rectangular dielectric resonator antenna array. For the 24-channel array, SNR in the center was slightly higher (1.32 a.u.) than the one for the 16-channel array (1.31 a.u.). Even though the peripheral SNR in the XY plane for the 24-channel array was reduced, additional SNR gains in YZ and XZ planes were observed. SNR along three different profiles (green—16-channel; red—24-channel) was compared

A 24-channel loop-dipole combined array (ε_r_ = 275; *d* = 0.25*b*) was simulated to investigate the feasibility of a multi-feed rectangular DRA for brain MRI at 7 T, and the scattering parameter matrix for the 24-channel array was evaluated (Fig. 9). For the adjacent elements: loop–loop coupling was -9.0 dB. Dipole–dipole (row 1) coupling was − 14.8 dB and − 14.7 dB (row 2). Dipole–dipole coupling between adjacent rows (1 and 2) was below − 20 dB. Loop-dipole coupling was − 18.8 (row 1) and − 18.9 (row 2). SNR for the 24-channel array in the center of the spherical phantom was almost identical (slightly higher) compared to the 16-channel: 1.32 vs 1.31 (a.u.). Additional dipole antennas enabled an apparent SNR increase along profiles in the XZ and YZ planes (Fig. 10).

**Fig. 11:**
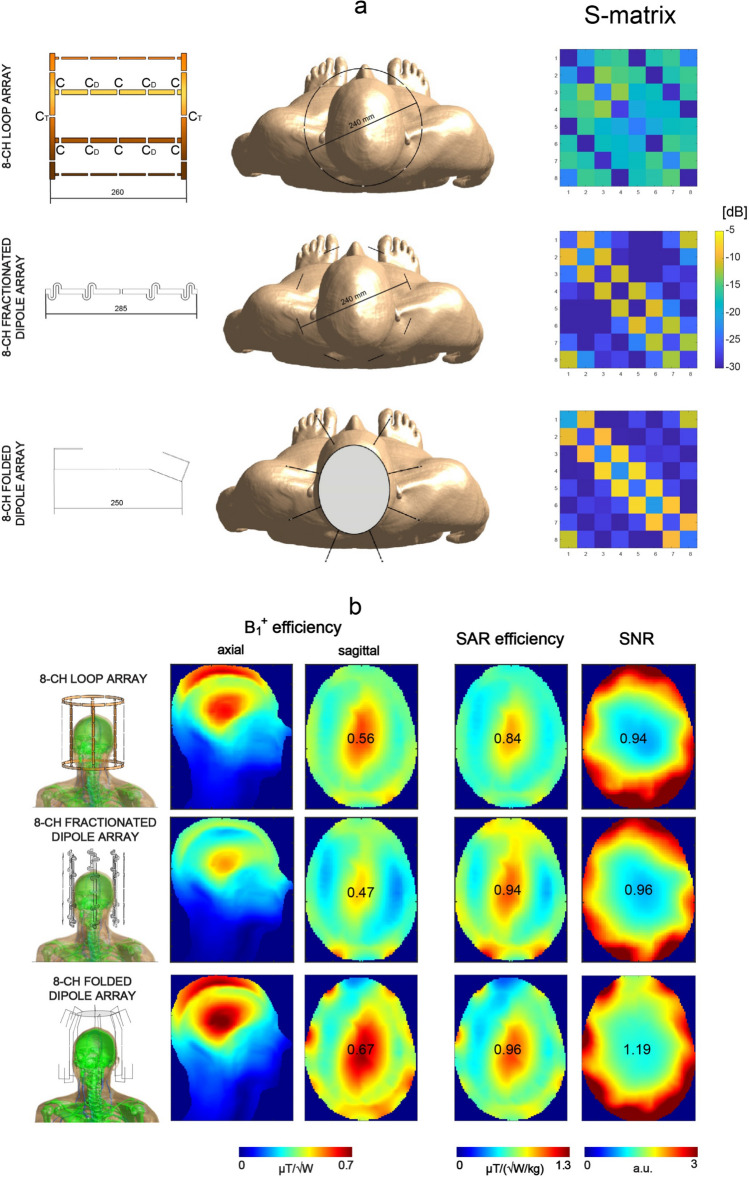
B_1_^+^ efficiency, SAR efficiency and SNR in the human voxel model Duke (central XY plane) for three reference array designs (see Methods). The highest transmit performance along with the highest central SNR was found for the close-fitting 8-channel folded dipole antenna array. Despite higher peak B_1_^+^ in the center of Duke’s head for the folded dipole array, an average B_1_^+^ (and B_1_^+^ homogeneity) across the whole axial slice was higher for the loop-coupled DRA arrays from Fig. 8 (see “Discussion”)

For the reference arrays, an average B_1_^+^ efficiency across the central axial slice was estimated (Fig. 11); it was found to be the highest for the close-fitting 8-channel folded dipole antenna array (0.42 ± 0.09 μT/√W; peak B_1_^+^ in the center = 0.67 μT/√W) followed by the 8-channel loop coil array (0.37 ± 0.06 μT/√W; peak B_1_^+^ in the center = 0.56 μT/√W) and the 8-channel fractionated dipole antenna array (0.32 ± 0.06 μT/√W; peak B_1_^+^ in the center = 0.47 μT/√W). SAR efficiency was calculated based on the pSAR_10g_ values obtained for each array: 0.49 W/kg (folded dipole), 0.44 W/kg (loop coil) and 0.25 W/kg (fractionated dipole). The 8-channel folded dipole antenna array provided the highest SNR in the center (1.19 a.u.) when compared with the other two designs: 0.94 a.u. for the 8-channel loop coil array and 0.96 a.u. for the 8-channel fractionated dipole antenna array.

## Discussion

This work demonstrates for the first time that a multi-feed, loop-dipole combined approach can be used to substantially enhance transmit and receive performance of rectangular dielectric resonator antennas for human brain MRI at 7 T. In this approach, loop elements should be used as DRA feed structures instead of dipole antennas to achieve higher B_1_^+^ and SAR efficiency, while combined loops and dipoles should be the most suitable in the receive mode to obtain the highest SNR.

In this study, loop elements and dipole antennas were combined in different configurations and used as RF multi-feed for rectangular dielectric resonator antennas (DRA). It was found for both single element and multi-channel arrays that the loop-only coupling scheme provided the highest B_1_^+^ and SAR efficiency in the center of a spherical phantom and yielded values significantly higher than those obtained with the dipole-only coupling scheme (Figs. 2, 5, 6). The data indicate that the loop-only can also outperform the loop-dipole in TX mode. This, however, needs to be interpreted with caution since in the case of the loop-dipole feed, both elements were always driven in-phase; additional phase optimization could potentially bring further transmit efficiency gains and it can be considered a limitation of this study. The data indicate that the dipole-only is not an optimal type of RF feed for obtaining the highest B_1_^+^ and SAR efficiency in the center of spherical-like samples designed to simulate the average human head. Nevertheless, dipole antennas were found to play an instrumental role in the SNR gain in the receive mode. By combining loops with dipole antennas, a significant SNR increase in the center of the spherical phantom was observed for all of the DRA arrays which were investigated in this study (Fig. 7). To summarize our findings, the loop-only coupling scheme should be used in the transmit mode to achieve the highest B_1_^+^ and SAR efficiency, while the loop-dipole should be the most suitable in the receive mode to obtain the highest SNR in spherical samples similar to human head in terms of size and electrical properties. To explain significant differences in B_1_^+^ efficiency between the loop-only and the dipole-only feed, we investigated the power absorbed by individual dielectric blocks (all 12). It was found that for the dipole-only feed, a substantial % of power was absorbed by the block, while it was not the case for the loop-only (59.5% vs. 25.6% averaged for all 12 single blocks from the study). Dipole antennas are radiating structures; the data indicate that in the case of dipole antennas, which are in direct contact with a dielectric medium of non-negligible conductivity, a substantial amount of RF power can be dissipated within the medium itself, thereby affecting B_1_^+^ efficiency.

The single-block data (Fig. 2) showed that none of the loop-induced, higher-order TE modes (*m* ≥ 3), which were observed, improved the DRA’s transmit performance compared with ${TE}_{11\delta }^{z}$ and ${TE}_{21\delta }^{z}$. Therefore, we conclude that those two TE modes can be considered the most promising for 7 T-MRI of spherical-like samples which are similar to an average human brain. Among all of the analyzed 16-channel rectangular DRA arrays, the ones with ε_r_ = 275 (*d* = 0.25*b*) and ε_r_ = 175 (*d* = 0.5*b*) provided the highest B_1_^+^ efficiency in the center of the spherical phantom (loop-only). By analyzing electromagnetic field patterns within these two blocks, we conclude that it is a higher-order, loop-induced ${TE}_{21\delta }^{z}$ mode (two EM field maxima were present along the z-axis) which should be associated with this effect. Also, the DRA with ε_r_ = 175; *d* = 0.5*b* (${TE}_{21\delta }^{z}$) provided the highest SNR in the center of the spherical phantom (loop-dipole). On the other hand, SAR efficiency was found to be the highest for larger DRAs with lower values of ε_r_: 75 (*d*/*b* = 0.75), 100 and 150 (${TE}_{11\delta }^{z}$). The data indicate that ${TE}_{21\delta }^{z}$ mode could be optimal to achieve the highest B_1_^+^ efficiency (loop-only) and SNR (loop-dipole) in spherical-like samples. Note, however, that this conclusion does not have to hold true for dielectric blocks with different dimensions with respect to the sample size (especially if dimension *a* would be significantly smaller than the one used in this work). The SNR gain observed for the loop-dipole combination can be explained by referring to the ideal current pattern theory developed by Lattanzi et al. [35], and which was verified experimentally at 7 T by Wiggins et al. ([36], in a cylindrical phantom), Erturk et al. ([37], for human body), and recently by Avdievich et al. ([38], for human brain).

The dimensions of the coupling loop as well as the distance between the loop and the rectangular block were kept constant in our study. We followed what was indicated by Aussenhofer and Webb, that too large coupling loops would change the boundary condition on the coupling plane of the DRA [13]. We do not expect changes in the coupling loop’s dimensions to bring considerable performance gains. When a DRA is designed to work as a resonator, and the resonance for a given mode can be measured at the bench using a pick-up loop connected to a network analyzer, the distance between the loop and the rectangular block must be adjusted experimentally to obtain the critical coupling between the loop and the resonator. In our study, that distance did not have to be adjusted as the DRAs were designed to work off-resonance. This does not change the fact that different types of modes, which played a crucial in the DRA’s transmit performance, were excited by a loop element placed at the fixed position.

Note that in this study, we investigated one particular orientation of the loop element with respect to the dielectric block and two different orientations of dipole antennas (top and bottom). Therefore, our findings should be considered limited to this particular positioning of loop-dipole elements with respect to the dielectric block. However, an alternative approach, i.e. placing loop-dipole elements on one side of the block instead of on the top of it, could be also considered useful for MRI applications. In such a configuration, the orientation of electromagnetic field vectors would be “swapped”, and the dipole antenna would act as a loop element and vice versa compared to the standard loop-dipole setting investigated in our work. In such a “swapped configuration”, a TE mode induced by a dipole antenna should provide more efficient transmit field than the one for the loop. This would be particularly apparent for larger rectangular blocks (*d* = 0.75*b*) with a dipole antenna placed on the top of the block, inducing an MR-inefficient mode [24]. If a loop element were positioned on one side of such a block, a similar effect (inefficient transmit field) could be observed, as for the dipole antenna placed on the top. So transmit performance gains observed in this work for loop-only coupling scheme can be explained by considering the interaction between a given TE mode (and its electromagnetic field orientation with respect to the load) and the load geometry. While doubtful, it remains unclear whether a loop-only coupling scheme for a rectangular dielectric block could provide higher B_1_^+^ efficiency compared with dipole-only when used for 7 T-MRI of deeper-located regions such as the prostate.

Three 16-channel rectangular DRA arrays (ε_r_ = 150, *d* = 0.5*b*; ε_r_ = 175, *d* = 0.5*b*; ε_r_ = 275, *d* = 0.25*b*) clearly outperformed the reference 8-channel bow tie array in terms of B_1_^+^ efficiency (1.48- to 1.54-fold), SAR efficiency (1.03- to 1.23-fold) and SNR (1.63- to 1.78-fold). The bow-tie antenna array was used as a reference because of the fact that it acts as a DRA (dipole-induced ${TE}_{11\delta }^{z}$ mode can be observed), and our goal was to show that our novel approach can be used to improve transmit performance of the state-of-the-art rectangular DRAs for brain MRI. Note, however, that the main purpose of the bow-tie antenna array was to be used both as an RF applicator (Thermal MR) and an RF antenna (brain MRI), and it was not optimized for providing good SNR performance for anatomical or functional MRI. Note that when the spherical phantom was replaced with the human voxel model Duke, the B_1_^+^ efficiency gain observed earlier for ${TE}_{21\delta }^{z}$ (ε_r_ = 175 and ε_r_ = 275) mode was not apparent anymore, and B_1_^+^ efficiency in the center of Duke’s head was for ${TE}_{21\delta }^{z}$ was similar as the one for ${TE}_{11\delta }^{z}$ mode (ε_r_ = 150 and *d* = 0.5*b*). This can be explained by examining the $\overrightarrow{H}$ field distribution within the dielectric blocks. ${TE}_{21\delta }^{z}$ mode largely benefited from the symmetrical geometry provided by the spherical phantom; the symmetrical distribution of two electromagnetic field maxima with respect to the curvature of the spherical phantom enabled more efficient RF power coupling. Unfortunately, this beneficial effect was reduced when the spherical phantom was replaced with Duke (asymmetry observed for the $\overrightarrow{H}$ field distribution inside the dielectric block), and the B_1_^+^ efficiency ${TE}_{21\delta }^{z}$ was similar to the one for ${TE}_{11\delta }^{z}$. This is not entirely surprising, since it was already shown that B_1_^+^ field distribution strongly depends on the interaction between given transverse electric mode and the geometry of the load [24]. Note that the simulations of multi-channel arrays did not include realistic models of tuning and matching circuits which are expected to negatively affect the absolute B_1_^+^ values presented in Fig. 8. The same applies to the necessary cable routing for all 24 channels which can further diminish observed performance gains. However, this remains to be fully verified once the prototype of the array is constructed. We still consider using the higher ε_r_ (and higher-order TE mode) of the dielectric block advantageous because it can provide higher SNR (Fig. 8). Furthermore, it allowed a decrease of the overall size of a single block, and increasing the total number of loop-dipole elements (up to 3 per block in this work, Fig. 9). Note that in previous studies, DRAs were fed using just a single element (either loop or dipole) per block (excluding orthogonal loops to induce HEM mode in volume resonators [11]). Our multi-feed, loop-dipole approach enabled using the total number of 24 channels instead of 8 as reported before. Moreover, for the 24-channel rectangular DRA, inter-element coupling was found to be very low and no additional decoupling methods were necessary (Fig. 9). In principle, a DRA does not have to be fed using maximally three loop-dipole elements. This is why we proposed the term “multi-feed” which refers to a broader idea: it assumes that the total number of loop-dipole elements, due to their intrinsically high isolation, could be further increased depending on the geometry of the block and its ε_r_. In principle, even without any modifications of the block’s geometry, there is enough space to add another dipole antenna per block to our 24-channel array. This idea can be further investigated in the future, since dipole antennas can be used as receive-only, and inter-element coupling is not that critical from a receive-only point of view. In future work, using a higher number of loop elements can be also considered.

An interesting feature of a dipole-fed DRA is its relatively low susceptibility to different loading conditions, which stands in contrast with small, inductively-shortened dipole antennas, which can detune by at least several MHz [39]. This makes a receive-only, dipole-fed rectangular DRA a promising candidate to be used in clinical settings when no patient-specific tuning prior to the examination is performed. Also, in this context, low inter-element coupling, which was found for the 24-channel DRA array, is particularly encouraging. Therefore, by exploiting these features, we envision the development of a head-adjustable, 8-channel transmit and 24-channel receive DRA array for brain MRI at 7 T. Such an approach could be a promising candidate for UHF-MRI applications involving e.g. electroencephalography (EEG) combined with functional MRI. Recently, RF safety for EEG-fMRI using 7 T-MR-compatible EEG electrodes was investigated [40]. In our DRA array design, there is a sufficient space at the apex of the head for EEG wiring. Also, a head-adjustable (and/or slidable) design can be used to facilitate direct access to the EEG electrodes when the subject is already on the scanner bed. This approach could provide an edge over traditional transmit/receive loop coil arrays which, mainly due to decoupling circuits (overlapping, capacitive or inductive networks), cannot be freely moved in space. Moreover, in such arrays, transmit/receive elements of are positioned rather far away from the head (in recent work, the diameter of the commercial coil was 28 cm) resulting in decreased transmit/receive performance. In this work, we focused on evaluating the performance of multi-feed, loop-dipole combined rectangular DRA arrays in the center of the brain. Therefore, our approach can be particularly interesting for EEG-fMRI in deeper located regions, e.g. brainstem [41]. Note, that, even though we focused on the center of the brain, a significant qualitative SNR gain in the periphery can be also observed for the loop-dipole approach when compared with dipole-only and loop-only (Fig. 7).

To benchmark the DRA performance against more conventional approaches, an 8-channel loop coil array, an 8-channel fractionated dipole antenna array and a close-fitting 8-channel folded dipole antenna array were designed and simulated (Fig. 11). The data indicate that the multi-feed DRAs can be still used as more efficient transmitters and receivers compared with the first two designs (loop coil and fractionated dipole). Note, however, that an increase in B_1_^+^ homogeneity for the loop coil array (0.37 ± 0.06 μT/√W) and for the fractionated dipole antenna array (0.32 ± 0.06 μT/√W) was observed. Simulations also revealed that all three loop-coupled DRA arrays (Fig. 8) can provide higher, whole-slice averaged transmit efficiency when compared to the 8-channel folded dipole antenna: 0.44 ± 0.10 μT/√W (ε_r_ = 275), 0.45 ± 0.09 μT/√W (ε_r_ = 175), 0.44 ± 0.09 μT/√W (ε_r_ = 150) vs. 0.42 ± 0.09 μT/√W (folded dipole). Note, however, that B_1_^+^ efficiency in the center of Duke’s head was the highest for the folded dipole array (0.67 μT/√W) compared to the loop-coupled DRA arrays (0.60 μT/√W for ε_r_ = 150 and 175). SAR simulations showed that pSAR_10g_ for the 8-channel folded dipole antenna (0.49 W/kg) was higher than for the DRA array with the highest SAR efficiency (i.e. 0.38 W/kg for ε_r_ = 150). In terms of SNR, the loop-dipole coupled DRA arrays can provide up to 2.5-fold higher SNR in the periphery of Duke’s head than the 8-channel folded dipole antenna, but the central SNR was found to be slightly lower (~ 6%). This effect can be related to the difference in performance at greater depths between short and long dipole antennas [33]. Note that the idea presented in this work can be further advanced and we envision several ways to do it. The 8-channel folded dipole antenna array was simulated with an elliptical shield which is known to have a beneficial effect on its transmit efficiency; there was no RF shield considered in the case of DRA arrays, and this can be an interesting direction for future research. Also, we assumed loss tangent to be constant throughout the whole study what could be considered one of its limitations; further SNR gains can be potentially achieved by reducing losses in the DRAs (more relevant for dipole antennas). Furthermore, the geometry of each DRA can be modified such that it follows a similar profile to the one defined by a single folded dipole antenna; this would result in an improved whole-brain coverage, and also enabling using a higher number of RF feeds. We also believe that the multi-feed approach can be a promising strategy in the context of future RF developments at higher magnetic field strengths as 10.5 T or 11.7 T (e.g. to considerably reduce the number of capacitors which are currently used to segment loop elements in large arrays).

Recent progress in materials engineering provides new opportunities for researchers who would like to use custom-tailored dielectric structures to enhance performance of the antennas for high field MRI. Ceramic materials can be currently developed in an impressively wide range of dielectric constants (up to several thousands) and low electrical conductivities, and this should provide a motivation to explore such structures in the context of novel RF concepts at different magnetic field strengths [42–45].

To conclude, a multi-feed, loop-dipole combined approach can be used to substantially enhance transmit/receive performance of multi-channel rectangular dielectric resonator antennas. This work provides novel insights into the rectangular DRA design for high field MRI and paves the way for the development of a new generation of multi-channel rectangular DRA for human brain MRI at 7 T.

## Data Availability

The data that support the findings of this study are available from the corresponding author, [D.W.], upon reasonable request.
